# An Ecosystem Model of Small and Medium Sized Enterprises Publisher ‘Tiers’: Publisher Size, Sustainability and Cultural Policy

**DOI:** 10.1007/s12109-021-09811-y

**Published:** 2021-05-25

**Authors:** Claire Squires, Helena Markou

**Affiliations:** Stirling Centre for International Publishing and Communication, Division of Literature and Languages, University of Stirling, Stirling, FK9 4LA UK

**Keywords:** Cultural policy, Independent publishing, SME publishing, Tiers model, Support models

## Abstract

This article establishes a quantitative and qualitative model of small and medium-sized enterprises (SME) publisher ‘Tiers’, in order to enable researchers and cultural policy makers to have a more granular understanding of the impact of publisher size. Through an aggregated set of case studies deriving from the UK, the article also develops an understanding of how to build a cultural support model for publishing based on publisher size, sustainability and company life cycle. What the Tiers model underpins in terms of cultural policy funding for publishers is a rigorous and developmental sense of a publishing ecosystem, offering a framework which is attendant to industry and broader contexts, and enables cultural policy funding to take into account publisher development, challenge and growth.

## Introduction

Publishing contributes substantially to the creative economy of nations; in the UK, from which this article draws its predominant data, it delivers turnover of over £6bn, directly employs 29,000 people, is the largest global book exporter, and underpins the economies of film, TV and theatre [28]. Publishing is, undoubtedly, big business in the UK. In the context of COVID-19, some publishers, particularly the larger conglomerate groups, saw turnover and profits increase in 2020 as lockdown circumstances pushed people towards increased reading [29, 41]. Individual publishers, however, and the sector as a whole, struggle to publish within certain sub-sectors in a financially sustainable manner, as an Arts Council England (ACE) report identified, and small, independent publishers are in an extremely precarious position, even before the economic pressures brought about by COVID-19 [1].

Nonetheless, publishing might be seen at the more commercial end of the creative industries, and as the Publishers Association’s (PA) statistics demonstrate alongside those of the Department for Digital, Culture, Media and Sport (DCMS), the publishing industry contributes substantially to the creative and broader economy of the UK, constituting approximately ½% of GVA [13]. However, for certain sectors of publishing, and for all SMEs (small and medium-sized enterprises), publishing is an extremely, and increasingly, precarious business. Its traditional business models demand long-term investment for future return. Changes in the market environment over the course of the twenty-first century (the rise of digital, demand from retailers for higher discount, downwards pressure on prices, the weak pound and the rise in costs, fewer sales and less value) have hit publishers particularly hard, even without the shock of COVID-19. Publishers also contend with the perennial issues of cash flow, generating back list income, returns, investment tied up in physical stock and warehousing costs. While many publishers are highly adaptable to circumstances, demonstrating ingenuity in the face of digital, market and pandemic-enforced change, and continuing to contribute to cultural diversity and artistic excellence, publishers find themselves in a time of increasing, and severe pressure. Outside of London and in the devolved nations of the UK, publishing is largely comprised of small and independent publishers, therefore making those areas subject to the additional challenges of achieving sustainable growth.

Given the exacting challenges for small publishers in particular, publishers are able to apply for funding within the devolved cultural policy landscape in the UK (e.g. publishers in England apply to ACE, in Scotland to Creative Scotland, in Wales to the Books Council of Wales, and in Northern Ireland to the Arts Council of Northern Ireland). With a variety of constraints, publishers can draw on public funding to mitigate against market challenges and difficulties, and to pursue initiatives in support of artistic excellence and audience reach. And yet, one of the questions around funding support for publishing is that some sub-sectors are—as the opening statistics presented by the Publishers Association suggest—profitable, comprised of big businesses as well as small. How, then, might cultural funding agencies make decisions around where to place funding based on an analysis which—while including the typical drivers of artistic excellence and diversifying audiences (see [34]), might also be informed by an understanding of publisher size?

In this article, we argue that an understanding of publisher size, and the potential—or otherwise—to weather the recurrent as well as unexpected economic challenges, should be central to decision-making around cultural policy funding for individual publishers and the broader publishing ecosystem. The article also has as a central purpose the establishment of a quantitative and qualitative model of SME publisher ‘Tiers’, and thereby seeks to make recommendations about where cultural policy investment should be oriented. In so doing, the article both lays clear a framework for understanding publisher development, challenge, and growth, as well as develops an understanding of how to build a cultural support model for publishing based on publisher size, sustainability and company life cycle.

Following a review of relevant literature relating to cultural policy (particularly literary cultural policy) as well as to small/independent/SME publishing, and the research methodology, the article examines the macro environment for publishing (generally, and with specific reference to Scotland and the UK), before moving onto the findings from the primary research, the proposal of the ‘Tiers’ model, and conclusions and recommendations.

## Literary Cultural Policy and SME Publishing

Although there is substantial scholarly literature on the broader frameworks for cultural policy generally, and cultural policy decision-making more specifically (for example see overviews from O’Brien [27], Bell and Oakley [2], discussions of literature and publishing within a state-delivered and cultural policy framework are more limited, beyond those relating to public libraries, censorship and copyright. Within the UK environment, an historical account comes with Asha Rogers’ *State Sponsored Literature: Britain and Cultural Diversity after 1945 *[34], along with details of Arts Council sponsored book events within D J Taylor’s *The Prose Factory: Literary Life in England Since 1918* [44]. Longworth provides an overview of the book in relation to the state and civil society, particularly with regard to literary development agencies [21]. In relation to the realm of foreign policy, and literature incorporated into the operations of soft power, studies include those of UNESCO’s books-based policy [4, 18] and that of the CIA in Africa [8]. Literature as an agent of the broader trends of regeneration through culture has also been addressed [3]. Policies and strategies relating to reading development, often at regional or city level, have also been produced [11]. Several of the articles in the co-edited special issue BOOK COMMERCE BOOK CARNIVAL of the journal *Memoires du Livre Studies in Book Culture* [9] focused on cultural policy interventions in terms of literary translation, location-based literary marketing and cultural interchange relating to book fairs and festivals (see in particular [12, 16, 26]). Audrey Laing [20] has recently offered policy recommendations to support the independent bookselling sector in Scotland. Within the specific context of one (perhaps rather exceptional) nation, Helge Rönning and Tore Slaatta’s *The Tools of Literary Politics: The Norwegian Model* [35] assesses cultural, economic and legislative policy in relation to literature, while Sapiro [38] surveys the production of cultural in late twentieth century France between the strictures of the state and the market. An overview of cultural policy in relation to literature has most recently appeared in Simone Murray’s *Introduction to Contemporary Print Culture: Books as Media* [23: 70–87].

What this article does, as detailed above, is to add to that growing literature by proposing a model of publisher size, in order to inform practical decision-making around cultural policy funding. As such, its approach is pragmatic, albeit underpinned by an understanding of the literature discussing small and independent press publishing.

Practitioner, scholarly and general accounts of contemporary publishing frequently settle on a normative, and oppositional, account of the literary marketplace. Global conglomerate publishing is perceived as in opposition to small and/or independent publishing, with the former articulated as both dominating the marketplace and itself dominated by the forces of capital, their shareholders, and ‘the market’; whereas independents or small press publishers are constructed as saviours of the uncommercial, the literary, and of ‘culture’ (examples of scholarly accounts include [22, 32]). A practitioner example of this line of argument is articulated by Susan Hawthorne in *Bibliodiversity: A Manifesto for Independent Publishing* [15], in which she asserts that independent publishers provide ‘a way of engagement with society and methods that reflect something important about the locale or niche they inhabit’ (xi). The International Alliance of Independent Publishers state that ‘“independent publishers guarantee the multiplicity and circulation of ideas, and as such are the real players and defenders of this cultural diversity within publishing”’ (cited in Hawthorne [15: 53]). The 2017 ACE report described independent publishers ‘as talent development agencies for authors at the more literary and experimental end of the scale [as…] a critical part of the UK’s literary infrastructure’, not least because ‘they tend to be based outside of London and therefore provide a conduit for local, traditionally under-represented voices’ (5).

Many of the scholarly accounts are underpinned by a Bourdieusian understanding, in which oppositional and hierarchical understandings of literary production are pre-eminent, and small and independent publishers are perceived and/or rhetorically constructed as autonomous (e.g. [24, 25, 43]). While such accounts are frequently nuanced (for example, Noël’s productive argument for ‘independence’ as a ‘polysemic’ term [24: 14]), these scholarly accounts follow the stratifications inherent in the global book business. Yet such depictions of the literary marketplace, whether deriving from academic, cultural policy and other forms of grey literature, trade journals, general media, or blogs, have a tendency to stereotype small press or independent publishing, creating, as one of us argues elsewhere, a skewed understanding of what a more varied small and independent publishing sector is attempting to achieve, what the business operations and motivations of such publishers are, and of how they fit into a broader understanding of a literary ecology [42].

This article also builds on Squires’ argument with Padmini Ray Murray in ‘The Digital Publishing Communications Circuit’ [36] that, rather than the ‘parallel universe’ of conglomerate and independent publishers conceptualised by John B Thompson [45: 155], there is rather a ‘complex ecology […] in which publishing companies, large, small and in between, have contrasts and similarities, and also tangible points of operational contact’ [36: 10]. In pursuing this line in ‘The Passion and Pragmatism of the Small Publisher’, Squires further argued that ‘the difference between a mid-sized company, operating with significant overheads, staffing levels and list sizes, is at least as far from a small, owner-publisher company as a mid-sized company is to a conglomerate’ [42: 215]. This article, then, seeks to answer the call at the conclusion to Squires’ previous work: that while the independent sector might indeed ‘provide a necessary corrective to the often more financially driven conglomerate sector’, ‘a more holistic sense of the multiple market sectors into which independent companies publish would provide a greater understanding of what it means to be a small publisher’ [42: 215﻿] and hence—as this article argues—of where cultural policy interventions might best be oriented.

Nonetheless, accounts of the challenges of publishing deriving from cultural policy spheres articulate the financial and business difficulties of specific types of literary production: the ACE report of 2017, for example, focused in on the challenges of producing literary fiction. This current article seeks to add a business-oriented understanding of small and independent publishing which, we propose, will be actively helpful in terms of generating more nuanced understandings of publishing. Such understandings will deepen comprehension of market sectors by not always focusing on questions around perceived literary excellence, genre, and the more literary end of the market. Rather, the understandings generated by the creation of a model of small publishing will have utility in terms of testing out and deciding on cultural policy interventions. Such understandings are both quantitatively and qualitatively developed, as the next section, which details the methodology undertaken in the development of the ‘Tiers’ model, underlines.

## Methodology

The ‘Tiers’ model described in this article was developed within the context of the macro environment for publishing in Scotland and the UK, detailed in the next section. The description of this context was derived from grey literature and book trade publications, and via primary research undertaken with a series of small publishing companies, using qualitative (semi-structured interviews and contextual industry publications and information) and quantitative data (book sales information, cash flow, balance sheets and data on funding awarded by Creative Scotland). The primary research was carried out during a period of research consultancy focusing on Scottish publishing market, undertaken on behalf of Publishing Scotland (the members’ association for publishers in Scotland, consisting of c70 publishers as well as network members), and also presented to Creative Scotland (the arms-length cultural policy funding body in Scotland, which directs governmental and lottery funding into arts organisations and creative businesses, as well as to individual cultural practitioners).^1^ The research consultancy project, undertaken before COVID-19 hit in 2020, was tasked to investigate sales trends and income in Scottish publishing, to capture and analyse datasets in order to evidence the challenges of the sector, and to set out recommendations and options for support mechanisms to build sustainability within it.

In order to do so, the research consultancy drew on a sample set of ten independent publishers based in Scotland, pre-chosen by the funder, as well as an analysis of the macro-environment. It did so in order to explore and analyse market conditions, challenges and opportunities for publishers operating in Scotland. It examined in particular how the book market operates for independent publishers, and through analysis of their business operations, broader sales trends and market shifts, sought to provide an understanding of the issues facing the building—and funding—of a sustainable publisher sector.

The sample set of companies were all trade publishers of fiction, non-fiction or children’s books. In the research consultancy, all the companies were anonymised, and the data gathered were not used in order to evaluate any particular company, its individual performance, or decisions about whether it should be funded or not. In other words, the purpose of the case studies was not to audit individual business, scrutinise their financial management, or make evaluative judgements about their operations. Rather, details of the anonymised companies were presented in order to demonstrate challenges and opportunities faced by *types* of publisher company (e.g. through the market sectors in which they operate; by the types of books they produce; and through their size and infrastructure). This detail was presented in order to enable understandings about the operations of publishing companies, and to facilitate decision-making processes around the orientation of the public funding of publishing, or indeed, other similar creative industries in Scotland. It is this latter information which is presented in this article.

The qualitative research included publisher interviews, conducted in 2018. The interviews lasted approximately between 60 and 90 min, and were all transcribed. The interviews were held either with the managing director or equivalent (the owner-publisher with the smallest of publishers), or in some cases with the financial director or equivalent. The interviews followed a semi-structured set of questions and topic areas, exploring the nature of the particular company (its output, infrastructure, staffing, history, and sales processes); the nature of the challenges facing the company including to financial sustainability and/or growth; the life cycles of its titles; and the conditions the company would like to see in the market to make publishing more viable. The interviews also asked publishers to discuss their own level of public sector funding in recent years, and asked for feedback on the ways in which the companies perceived public sector funding currently to be working, and how they might prefer public sector funding to operate in the future.

Quantitative data were derived from two core sources. Firstly, data were collected from the sample of Scottish publishers. These data included business level profit and loss (P&L) via a template spreadsheet, in which publishers provided the income, expenditure, administration and staff costs over a five-year period (2013–2017); as well as details of working capital, including the typical frequency of invoicing and payment terms for each type of income and expenditure to give an approximation of publishers’ working capital cycle. Secondly, trade consumer market (TCM) data were collected from Nielsen BookScan, in order to provide market sector overviews.^2^ In addition, title data for each publisher were analysed in terms of genre, volume, value, pricing, discounts and number of authors.

## Publishing Contexts and the UK and Scottish Publishing Markets

In order to set the Tiers model in context, this section offers an overview of first the UK, and then the Scottish, publishing markets. The latter is nested within the former, but its environment is also differentiated due to being populated largely by small and independent publishers, a devolved cultural policy environment, as well as cultural and political differentiation which affects content creation [19]. The overview also illustrates some generalities of the (traditional, largely print-based) publishing business model.

The book publishing industry is generally considered to have five major market sectors: trade or consumer; academic; educational; professional; and science technical medical (STM). Broadly speaking these sectors have quite different products, sales trends, and routes to market. In turn, they have evolved distinct production calendars, product life cycles and sales infrastructures. Nevertheless, there are common factors that span across all sectors; in economic terms publishing activity can be seen as an investment of time, money and human resources in developing content and making it public. Capital is invested upfront in both the print production of physical stock and the development of digital products, with no certainty of a return on those investments. This up-front capital investment is an intrinsic part of the risk aspect of the traditional publishing model. It means that publishers have substantial amounts of their cash tied up in stock, which they must also pay to be warehoused or otherwise stored. Developing models (e.g. crowdfunding) can mitigate or take away completely the risk of traditional publishing, as can short-run digital publishing. Developing models and technologies (e.g. crowdfunding, short-run digital printing and digital-first publishing) lower the financial barriers to entry and dramatically reduce, or negate, the capital expenditure on variable costs (i.e. those that increase with scale of output) required to manufacture print products. However, offset litho printing in terms of per-unit cost is still substantially cheaper than short-run digital printing, meaning the former allows economies of scale [39: 137]. Moreover, none of these newer models and technologies completely mitigate the financial risk of publishing because the fixed costs of bringing products to market (editorial, design and origination costs) remain irrespective of the number of copies printed.

Trade publishing generally refers to the fiction and non-fiction books sold to consumers through bookshops and other non-specialist retail outlets such as supermarkets, museums and garden centres, and is the focus of attention for cultural policy funding, and hence this article (although educational books also have a clear relationship to state policy, including schools curricula, purchasing and procurement policy). The sector is further distinguished by the split between frontlist and backlist (new titles and old titles), and the fact that it may be swayed by ‘black swan’ events such as bestsellers [7: 93].

Beyond these aspects, the consumer book market is defined by its level of discretionary purchasing, which has the effect of promoting competition [14: 52–3]. A significant proportion of trade titles are bought for leisure or entertainment, rather than necessity, and benefit from impulse purchasing [39: 49]. Books are not just competing against other books, or even other forms of cultural activity (such as theatre, music or visual arts), they are competing for attention against *all* leisure and entertainment activities (such as sports and eating/drinking out).

Traditionally, bricks-and-mortar retailers must balance the constraints of shelf space when selecting the range of stock against the size of the local population. When shelf space is finite, it makes economic sense for retailers to focus on the titles that sell in the highest volume, i.e. those with the broadest mass-market appeal. Therefore, backlist titles with a proven track record typically account for the vast majority (between 60 and 80%) of the stock displayed by physical/high street bookshops.^3^ Publishers that have a backlist of proven titles can use the revenue to support business growth and the development of new titles. This business model necessarily advantages established publishers; start-up publishers take time to develop a backlist which is not normally available to them within their first few years.^4^

A publisher’s backlist can provide substantial benefit to a company, but again it demonstrates another risk in publishing: that of cashflow. Publishers invest upfront, but frequently do not make any returns for over a year. Additionally, a company cannot be built upon one title, no matter how successful it is—or appears—to be. Growth is enabled by having a list, a number of titles that can create growth. Cash flow is a major challenge, as upfront costs and ongoing expenditure on stock-holding and infrastructure (e.g. office space, warehousing, IT hardware and software, sales representation and marketing services, whether in-house or external) need to be made well in advance of any revenue generation.

The inherently risky and necessarily long-term nature of the publishing business means that drawing down start-up capital is particularly hard for new publishers, making it one of, if not the biggest, inhibitor of new publishing companies. New publishing companies, as Clark and Phillips detail, ‘usually take at least four years from scratch to turn a profit’ [7: 9]﻿. Without alternative forms of income—private funds (from those who can afford it; there are inevitable equalities issues,^5^ or some other form of external funding, including public sector funding—it can be extremely difficult for start-up publishers to establish and grow viable businesses.

Newly published frontlist titles require a significant push from sales representatives and substantial marketing and promotional activity for two reasons. This sales and marketing push is needed firstly to persuade booksellers to stock them, and secondly, to sell in such quantities that they remain on the bookshop shelves for the medium to long term. The peak period for fiction sales is within three months of publication—sometimes only a few weeks—meaning the time allowed by retailers for a title to fail or succeed is very short [7: 93]. A further peculiarity of the UK book trade is that stock is usually only ordered by retailers on a sale-or-return basis. Sale-or-return agreements mean that an overestimation of the potential sales of a book can result in unsold stock being returned to the publisher, with the retailer requiring credit. The system mitigates booksellers’ risk and encourages them to stock widely, but it places all the risk in the publishers’ hands [39: 199]. Receiving unexpected returns can be highly injurious—even potentially life-threatening—to a publishing company.

In the twenty-first century, publishers have also seen the discounts required by booksellers rising. Many publisher-bookseller deals are subject to deep discounting, which means that well over half the cover price, or RRP (recommended retail price) is given to the bookseller. Discounts can sometimes rise up to almost 70% [39: 199]. In addition to deep discounting, rising direct costs (including of print and production), in line with inflation, and in the context of a weak pound post-EU referendum in 2016, mean that publisher revenue in the UK has been substantially squeezed. Publisher average selling prices (ASPs) have dropped in real terms in recent years (ACE reported in 2017 down 44% for hardback fiction since 2001, down 25% for paperback fiction), meaning that publisher gross margins are being adversely affected (12). This trend began after the demise of the Net Book Agreement (NBA) in the mid-1990s, but has exacerbated over the course of the twenty-first century, as retailers promote heavily on price. This can mean that even if publishers are actually selling more copies in volume terms than in previous years, the value derived from them is flat, or even—in real terms against inflation—decreased. It is becoming harder and harder for publishers to stand still, let alone grow.

The 2017 ACE report further illustrated the challenges of the market for literary publishing in the UK. The overall findings of the report clarified the extremely difficult trading conditions for writers and publishers of literary fiction, including the diminution of review space and a shrinking number of bookshops (27, 19). Among its key findings were the falling rates of print sales for literary fiction, the fall in price in real terms of literary fiction titles (meaning less money is received by publishers per copy), and—while ebook sales have risen in other sectors of the book market (e.g. in genre and commercial fiction)—literary fiction has not seen such a compensation for falling print sales (3). These findings mean that the ‘market for print books has shrunk’. Moreover, positive soundings on gains in value in the overall market are *not* replicated in the market for fiction, ‘which remains flat in absolute terms and continues to decline in real terms’, making literary publishing ‘exceptionally tough’. This is ‘emphatically not an easy time’, concludes the report (11, 15, 24, 4).

Publishing, then, may be thought of as an industry that comes with substantial financial risks associated with managing its many individual products. Being a large publisher with a healthy backlist is advantageous in terms of managing overall risk, and being a smaller, newer one is disadvantageous. The window of opportunity for new titles to succeed is short. The sales and marketing capacity of a publisher is critical to the core objective of making content public and reaching a target market.

The site of the primary research contributing to this article, Scotland, is particularly affected by the challenges of financial risk relating to size and longevity. The publishing industry in Scotland predominantly consists of small and medium-sized independent publishers, although there are also offices of global conglomerates and publishing activity within larger organisations. Indeed, small and independent publishers have a particular role in discovering, nurturing and supporting authors, and this is crucially important in sustaining a vibrant national culture of writing, reading and publishing, as discussed above. Since 2010, independent Scottish publishers have, for example, published novels winning, longlisted and shortlisted for the Booker Prize and International Booker Prize; have expanded the reach and range of children’s books by Scottish writers; celebrated the centenary of Muriel Spark; launched the careers of novelists, short story writers and poets; enabled the publishing of books in Gaelic and Scots; introduced into the Anglophone market writers in translation from around the world; and moved beyond a crowdfunded, start-up phase to a concerted publishing programme. Much of this activity has been supported in part by funding from Creative Scotland or other cultural policy agencies, and much of it would not have happened, certainly to such a degree, without that funding.

More broadly, there is an energetic literary scene in Scotland, including its literary festivals and opportunities afforded to writers and readers via Scottish Book Trust’s various schemes (e.g. Live Literature; Book Week Scotland; the First Minister’s Reading Challenge). However, despite its clear successes and contributions to the cultural and economic life of the nation, and to spreading culture beyond Scotland’s borders, the Scottish publishing industry faces significant challenges in the face of current market conditions, even before the financial shock of 2020’s global pandemic.

In addition to the general problems of the (economic) devaluing of literary fiction in the UK England market, Scotland’s publishers suffer from the centralisation of the UK publishing industry, which is heavily based in the south-east. As the ACE report puts it:

almost all literary publishing is concentrated on London. The major publishers are in London. All but one of the Independent Alliance are in London. The newspapers and reviews are based in London. Decisions are made in London. There is little sign that any of these things will change in future [1: 36]﻿

Although there have been recent movements to address this (e.g. announcements of publishers opening regional offices [5]), the issue of centralisation remains problematic. For all independents, wherever located, ‘poaching’ is also an issue, with their initial risk in investing in and nurturing writers then lost to larger publishers able to attract writers by larger advances and bigger marketing reach. For writers, this may be a positive as their careers grow, but for independent publishers, retaining successful authors on limited budgets is challenging. It has been suggested that larger publishers picking up authors initially published by small or independent publishers should pay an equivalent to a sports ‘transfer’ fee to the latter [6].

Digital technologies have presented independent publishers with opportunities: short-run digital printing; new funding models such as crowdfunding; and marketing via social media, for example. Indeed, the ACE report comments on new English independents established outside of London at the same time as consolidation has been occurring amongst the conglomerates [1: 23–24]. The challenge for these companies, as with Scottish independents, is how to sustain growth, to tackle the challenges of stock holding and cash flow in a devaluing market, and—if the company manages to sustain itself—to enable effective succession planning.

There is also the need to develop and sustain a scaled-up industry in order for the whole ecosystem not to be resting on very precarious ground, and—should individual publishing companies manage to emerge from COVID-19—to continue to develop. The Publishing Scotland submission to the Scottish Government’s Culture, Tourism, Europe and External Affairs Committee with respect to the impact of COVID-19 referred to a ‘a catastrophic drop in income over the past few months and extending into the summer. This impact has affected the entire publishing ecology of publishers, writers, illustrators, freelancers, bookshops, and other parts of the supply chain and infrastructure’ [30: 1]. In order to explore further how an understanding of publisher size and sustainability can contribute to both recovery and ongoing resilience, the next section turns to the model of SME Publisher ‘Tiers’.

## A Model of SME Publisher ‘Tiers’

This section unfolds the model of publisher ‘Tiers’, before going on to explain their utility in terms of understanding the nature of small publishers and their challenges, and then outlining how this model might enable cultural policy agencies to make decisions about where to make funding interventions. The mixed methods approach detailed in the methodology was used in order to develop the model, which is based on both quantitative and qualitative metrics. As one of us has detailed elsewhere [42], there is a high degree of variability even in the quantitative calculation of publisher size, using measures including the number of salaried employees, revenue, market share, or the number of books produced per year (either individual titles or units produced). The European Commission [10] defines company size through a combination of turnover and employees, dividing SMEs into ‘medium-sized’ (fewer than 250 employees/€50m), ‘small’ (fewer than 50 employees/€10m) and ‘micro’ (fewer than 10 employees/€2m). The PA takes a slightly more granular approach, but nonetheless relies on turnover and employee headcount. The Independent Publishers Guild (IPG), which represents publishers across the UK, defines a series of membership ‘bands’ based on turnover, moving from the lowest at up to £100k, up to £10m + [17]. Publishing Scotland’s subscription rates are similarly based on company turnover [31]. Both these classifications are used to determine a scaled level of membership fee, but do also stratify companies via a quantitative metric. What the proposed ‘Tiers’ model does is add both further quantitative measures, supplemented with qualitative information that enables a more holistic and contextual understanding of where cultural policy support could best be aimed, thereby leading to an ecosystem approach.

The individual publishers with whom research was conducted comprised a small but representative sample of independent publishing in Scotland. Given that the vast majority of the sample fell into the European Commission’s ‘micro’ category, to enable further analysis, we further subdivided the companies into four Tiers:

- Tier 1: Micro business of less than £100k turnover
- Tier 2: Micro business of £100k–£1m turnover
- Tier 3: Micro business of £1–£8m
- Tier 4: Small business of £8–£44m

The value in bringing together the sample publishers under these four Tiers is that some commonalities began to emerge in terms of their type and degree of challenge, which are in turn proposed as a starting point for establishing a granulated quantitative and qualitative approach to definitions of ‘small’ publishers. These Tiers are summarised in Table 1, and detailed below.

**Table 1:** Tiers 1–4 of micro and small publishers

*Tier 1: Micro business of < £100k turnover*
‘Kitchen-table’ publishers, i.e. operating from owners’ homes Minimal-to-no salaried staff, and inconsistent financial resources to afford staff Subsidised by owner’s unpaid time Personal finances invested by owner Challenge of unfavourable payment/credit terms from suppliers, and limited leverage to negotiate Without infrastructural support, hard to grow beyond current level; lack of control due to reliance on suppliers and freelancers Typical start-up dilemma of how to achieve growth and move into Tier 2 Entrepreneurial in nature Lifeblood of publishing industry
*Tier 2: Micro business of £100k–£1m turnover*
Greater level of infrastructure than Tier 1 companies, including offices Small number of salaried staff, and inconsistent financial resources to retain staff Challenge of infrastructural costs Challenge of attempts to grow Challenge of unfavourable payment/credit terms from suppliers and limited leverage to negotiate Companies struggle without cultural policy funding, other external investment or owner subsidy through work external to the business Normally, former Tier 1 businesses that have grown Challenge of becoming Tier 3 businesses
*Tier 3: Micro business of £1–8m turnover*
Typically, longer established than Tier 1 and 2 companies Operate on a more stable footing than Tier 1 and 2 companies Substantial offices and infrastructures (in small business terms) Higher number of salaried staff Much less precarious in terms of continuing operations Typical challenges include cash flow, discounting, returns, reaching geographical markets beyond the immediate region or small nation, issue with dealing with export markets, including currency exchange Challenge of financial risks being larger in absolute terms (bigger print runs/larger orders/higher risk of large returns) Cultural policy support beneficial, but can also come in the form of macro-level infrastructure
*Tier 4: Small business of £8–44m turnover*
Operate at a much greater level of staffing than Tiers 1–3 In addition to book sales revenue, companies derive substantial income from export and rights sales, and through editorial and acquisitions strategies Revenue derived from backlist as well as riskier frontlist income Challenges of keeping systems up-to-date

### Tier 1 Micro Business of Less Than £100k Turnover

The Tier 1 companies, who might commonly be termed ‘kitchen-table’ publishers, typically operate from their owners’ homes, with minimal-to-no salaried staff, and inconsistent financial resources to afford staff. Companies in Tier 1 are likely to be subsidised to varying degrees by their owner’s unpaid time, and sometimes by the owner investing their personal finances into the company. They have the challenge of unfavourable payment and credit terms from suppliers, and limited leverage to negotiate. Tier 1 companies often demonstrate ambition to develop their infrastructure, but without investment in infrastructure it is hard to envisage them growing into the size of companies at the subsequent Tiers. Among the specific companies surveyed, one had benefited more from cultural policy funding, but was also working in a market sector that has both higher costs and smaller markets.

Without infrastructural support from cultural policy funding or elsewhere, it is hard to envisage Tier 1 companies being able to move beyond the level at which they currently operate. Tier 1 companies face a typical start-up dilemma of how to achieve growth. The entrepreneurial nature of Tier 1 companies, however, is also the lifeblood of the publishing industry, and is crucial to its renewal.

### Tier 2 Micro Business of £100k–£1m Turnover

Tier 2 companies have a greater level of infrastructure than Tier 1 companies, with offices, and a small number of salaried staff, albeit frequently with inconsistent resources to retain them. Such companies attempt to grow their businesses in a sustainable way, but still encounter a high degree of challenge, which in part comes from that attempt to grow and support infrastructural costs. Like Tier 1 companies, Tier 2 companies have the challenge of unfavourable payment and credit terms from suppliers, and limited leverage to negotiate. Tier 2 companies show a variety of ways in which they manage to continue to operate their companies, from support via cultural policy funding to providing income-generating publishing services work. Companies in this Tier, particularly in a small-nation context, are well recognised within their market sectors. With the specific sample, some companies benefited from additional income streams such as partnership publishing programmes which provided steady income, or from the successes and promotional visibility brought to trade book companies by literary prizes. The latter in particular had enabled company growth and profile, and vindicated editorial choices and decision-making.

Without either a substantial and continued degree of cultural policy funding or other external investment, or a degree of subsidisation through work external to the core business of the company, Tier 2 companies struggle. The surveyed companies in this Tier were all formerly Tier 1 companies. Beyond the challenge of sustaining their Tier 2 position, all the companies face the challenge of how to get into Tier 3.

### Tier 3 Micro Business of £1–£8m Turnover

Tier 3 companies are, on the whole, longer established than companies in Tiers 1 and 2, and operate on a more stable footing. They have a higher number of salaried staff, substantial offices and infrastructures (at least in independent publishing terms), and a much less precarious foothold on continuing operation—which reflects and develops from their longer histories of several decades of development. Typically, Tier 3 companies have healthy turnovers, and are effective at exploiting market niches. Nonetheless, companies in Tier 3 are presented with ongoing challenges typical to publishing overall and in this particular geographical sector: cash flow, discounting, returns, reaching markets beyond the local one, and issues with dealing with export markets including currency exchange. They also have the challenge of financial risks being larger in absolute terms (e.g. bigger print runs, larger orders, and a higher risk of returns).

Within the sample, the majority of companies in the Tier had benefitted from cultural policy funding to underpin their growth, and all discerned areas in which they as companies would benefit from more funding to grow further (although sometimes this support might come in the form of macro-level infrastructure).

### Tier 4 Small Business of £8–£44m

Tier 4 refer to companies which operate on a much larger scale in terms of staffing and turnover, falling into the European Commissions’ ‘small’ rather than ‘micro’ category. Businesses categorised as Tier 4 have clear advantages over those in lower Tiers. Typically, as well as revenue from book sales, they might derive substantial income from export and rights sales, and through editorial and acquisitions strategies growing revenue from backlist rather than being dependent on riskier frontlist income. Such companies might operate within highly competitive markets, with a high degree of attendant risk to that, including in directly competing with conglomerate publishers. In addition to operating within competitive markets, Tier 4 companies’ key challenges often reside in keeping systems up-to-date, and in decision-making around formal alliances and sales operations, in their further efforts to sustain and grow market position.

### Tiers Summary

It﻿ would be important to test the Tiers model against further empirical cases, given the sample size. Nonetheless, even from this small sample clear patterns started to emerge. Looking across all four Tiers, it is evident that there are significant challenges at all levels around perennial publishing issues (cash flow, discounting, returns, reach to markets outside the local region and internationally, frontlist/backlist mix, effective systems and control of metadata, selling books). Companies in Tiers 1 and 2 in particular demonstrate high levels of precarity, commitment and even braveness in running their companies. All companies can be very adversely affected by external circumstances over which they have little or no control (e.g. changes or issues with external agencies; exchange rates; external events which might render the promotion of a particular title inappropriate, such as a terrorist attack, or jeopardise the whole book-buying infrastructure, such as a global pandemic; and personal circumstances). Depending on the size and financial position of a company, these issues can create huge and potentially insurmountable challenges to a business, and in any single year turn a well-managed, carefully constructed and strategic operation to the red.

The analysis of the sample companies focused on the five years from 2013 to 2017, and therefore did not examine in any depth the development and growth of the companies prior to 2013, focusing instead on this brief longitudinal period. Nonetheless, there is a clear correlation between the maturity of the companies under analysis and their current financial stability and turnover, with newer companies largely being on a more precarious footing. The sample companies in Tiers 3 and 4 were in relative terms long-established. Notwithstanding substantial start-up funding or beginning as spin-out companies from other businesses, it could be assumed that all publishing companies pass through some iteration of Tiers 1 and 2 as part of their evolution before they can arrive at the less precarious positioning of Tiers 3 and 4. Publishing is a long-term business, and even a ten year-old company is youthful, given publishing’s ideal financial scenario in deriving significant income from backlist sales and exploiting intellectual property for the duration of copyright. Moreover, with some exceptions in the sample, the greater the company’s turnover, the greater its turnover to staff ratio (ie the company generates a higher turnover per staff member). A company is therefore more efficient as it becomes larger. Both these preliminary findings would suggest it is easier to be financially effective if the company is older, and larger (e.g. in Tiers 3 and 4).

## Conclusion and Recommendations

What, then, does the model of SME Tiers demonstrate, and in particular what is the model’s utility to cultural policy agencies when making decisions about where to focus funding? A clear challenge to funders is how to enable support for publishers as they go through and beyond a start-up phase of Tier 1, supporting them through the financial precarity and infrastructural investment of Tier 2, to an evolution into Tier 3 and 4. It is extremely unlikely that a publishing company can start its operations at Tier 3 or 4, or even arrive at those levels within its first 5 years, and—depending on the macro environment and choices of market sectors in which to publish—is only likely to arrive there with careful strategic growth and support from multiple sources.

Analysis of these sample publishing companies demonstrates a range of issues which at one level may benefit from ongoing and new collective infrastructural support, but which also have requirements that are very varied, depending on the publishers’ developmental stage. Although companies in Tiers 3 and 4 may have the financial and infrastructural stability to deliver on particular projects, including those which might have artistic excellence and/or principles of equalities, diversity and inclusion (EDI) at their core, any over-riding focus on them would not be conducive to sustaining and growing the sector as an ecosystem.

The research consultancy on which this current article is based did not seek to articulate how the cultural policy agency should structure its funding for publishers in the future, nor did it comment upon past funding models or decisions. Rather, the role taken was to demonstrate the range of challenges and opportunities facing a sample of independent publishers; to focus on particular recurrent areas of challenge; and to provide evidence in order for the cultural policy to make decisions about the future structure of its funding streams for publishers. The latter can only be done with a cultural policy funder’s own understanding of what it wants or is able to fund; which may or may not sit alongside what might be perceived to be a desired effect upon individual publishers, and upon the local publishing landscape more generally. In the specific terms of the consultancy project out of which this current article arose, the emphasis on the much greater need for sales management led to Publishing Scotland obtaining funding from Creative Scotland for a part-time post, which from late 2020 has provided coaching on sales support for small publishers, an intervention designed in order to build infrastructural capacity.

That said, this concluding section offers reflection and recommendations on how the Tiers might present a useful model around which to structure funding, bearing in mind publisher size and the challenges of sustainability, and proposes that the following key points be taken into consideration both by cultural policy funders, and by those seeking to understand and analyse how to build successful publishing ecosystems.

First, an effective publishing ecosystem needs to have business start-ups; developing companies; and established companies (e.g. Tiers 1–4, and ideally larger). Cultural policy funding should seek to contribute to that ecosystem. As a consequence, decisions around individual publishing companies should be made in relationship to publisher size and age, bearing in mind companies’ evolution, journey and trajectory. Such decisions should be made alongside an understanding of both the challenges of the publishing business model (cash flow; stock; sale or return), and the consequent need for companies to commit long-term investment in order to succeed, as well as the increasingly difficult trading decisions facing publishers over the course of the twenty-first century, particularly for those who are producing less commercial products, including literary fiction, and even more so in the wake of COVID-19.

Second, it is crucial to understand that many independent publishers, and as a consequence small-nation publishing ecosystems, exist in a state of precariousness and fragility, despite their public successes and evident contributions to regional, and international, cultural life. In order to bring sustainability to the core of cultural policy funding, it is important to consider whether publishers can ask for support for infrastructural costs rather than/in addition to project or title costs, and whether collective mechanisms and support for broader infrastructural organisations and operations (e.g. in relation to sales and marketing) can be considered as well as funding for individual publishing companies.

With regard to both conclusions, decision-making should be underpinned by a cognisance that an effective publishing ecosystem contributes to an even broader environment for literature, literacy, reading and writing; contributes to other cultural activity (e.g. adaptations into films, TV and theatre), education and information, and to the wider creative economy. What the Tiers model could underpin in terms of cultural policy funding for publishers is a rigorous and developmental sense of a publishing ecosystem. The Tiers model offers a framework which is attendant to industry and broader contexts, and enables cultural policy funding to take into account publisher development, challenge and growth.

## Footnotes

1 The research consultancy was undertaken in 2018 with ethics agreement that the individual company data – both qualitative and quantitative – and the resulting report, containing as it did financial data from the sample companies (albeit anonymised) was confidential. However, with agreement of the funder, this present article focuses on aggregated data and understandings derived from the research undertaken for the report, and thus provides for a much wider audience – both scholarly and practitioner – analysis, models, and recommendations.

2 There are limitations to Nielsen BookScan data. The dataset captures print book sales sold through a comprehensive panel of physical and online retail outlets within the UK. The panel includes Amazon, high street bookshops and non-specialist book retailers such as supermarkets, museums and garden centres, but excludes ebook sales, library sales and direct sales such as educational books sold in bulk to schools.

3 The significance of the backlist within the value chain is explored in greater depth by Markou in her forthcoming doctoral thesis at the University of Stirling, *The Shelf-Life of Books: An Exploration of the Lifecycle and Longevity of Books in the UK in the twenty-first century*.

4 An area which would benefit from further research in terms of start-up publishers is the time period over which it takes to build a backlist that can support a publishing company sufficiently for it to be a sustainable aspect of company growth.

5 Repeated research reports and academic work have indicated the issues with publishing’s lack of diversity and inclusion; see for example [33, 37, 40].
